# Community-Engaged Strategies for Recruitment of Korean Americans in Community-Based Research Studies

**DOI:** 10.1093/geroni/igab046.1823

**Published:** 2021-12-17

**Authors:** Haera Han, Hochang Lee, Miyong Kim

**Affiliations:** 1 Nursing, Baltimore, Maryland, United States; 2 University of Rochester, Rochester, New York, United States; 3 School of Nursing, The University of Texas at Austin, Austin, Texas, United States

## Abstract

With increasing numbers of researchers targeting ethnic minorities to address health disparities, it is important to address the unique needs of Korean American (KA) older adults—a “hard to reach” yet one of the most rapidly increasing ethnic and age groups in the nation. The purpose of this paper is to describe the main barriers to research participation and to identify facilitators for recruitment of older KAs. We have analyzed recruitment data pertaining to more than 10 community-based KA research studies we have conducted for the last ten years. There were a number of unique recruitment challenges in regard to the culture, language, and sociodemographic characteristics of the participants. Examples of effective recruitment strategies included: aligning the research agenda with the priorities of the community; establishing collaboration with ethnic churches and ethnic media; recruiting and training bilingual volunteers and community health workers; and placing liaison research staff in the community.

